# Bacterial translocation in acute lymphocytic leukemia

**DOI:** 10.1371/journal.pone.0214526

**Published:** 2019-04-01

**Authors:** Yajing Song, Peter Gyarmati

**Affiliations:** University of Illinois College of Medicine Peoria, Peoria, Illinois, United States of America; German Cancer Research Center (DKFZ), GERMANY

## Abstract

Bloodstream infection (BSI) is the major cause of mortality in acute lymphocytic leukemia (ALL). Causative pathogens in BSI originate from the gut microbiota due to an increase in intestinal permeability, a process known as bacterial translocation (BT). The gut microbiota in physiological conditions is controlled by a large number of immune cells as part of the gut-associated lymphoid tissue (GALT).The aim of the current study was to investigate the mechanism of bacterial translocation in leukemia by identifying and characterizing alterations in the GALT in leukemic mouse model. Our studies revealed a severe impairment of the GALT characterized by a loss of lymphatic cells in ALL, which eventually led to BSI. We identified differentially expressed genes in the intraepithelium and the lamina propria, which may contribute to BT and to the impairment of lymphocyte migration.

## Introduction

Hematological malignancies are the most common cancers during childhood, and leukemia comprises of 30% of all pediatric cancers. Acute lymphocytic leukemia (ALL) is the most frequently occurring pediatric leukemia, comprising 80% of childhood leukemias, with the incidence highest between 2–5 years of age [1].

Bloodstream infection (BSI) is a major cause of mortality in leukemias, due to the underlying disease and therapy-induced neutropenia [2–4]. The origin of BSI, at least in part, is bacterial translocation (BT) [5, 6] as a consequence of an increase in intestinal permeability.

The gut-associated lymphoid tissue (GALT) is the largest immunological organ in the body [7]. It is primarily located in the lamina propria (LP) [8] and also includes scattered lymphoid cells in the mucosal epithelium (intraepithelial lymphocytes, IEL) [9]. The highest number of immune cells in the body can be found in the GALT in order to ensure the integrity of the intestinal barrier against microbial translocation. The GALT also helps to maintain homeostasis with the gut microbiota to preserve its regulatory functions [6].

Although the “leaky gut” phenomenon is known in leukemias [10], its effect and the extent of its influence are controversial [11]. Additionally, the investigation of intestinal permeability in leukemias is often confounded by comorbidities [12] such as intestinal cancers and/or anticancer treatment, e.g., chemotherapy, as both damage the epithelial barrier [13, 14]. Moreover, permeability studies are typically based on the use of sugar molecules, but absorption cannot be directly interpreted for translocation of bacteria because of the lack of antigenic property [15].

The current study aimed to investigate the mechanism of BT in leukemia using a pediatric ALL mouse model. Our aims were to decipher structural and molecular changes in GALT, and to evaluate their contribution to the pathogenesis of BSI in leukemia.

## Materials and methods

### 3.1 Mice

Three weeks old female Nod/Scid mice were used in this study (Jackson Laboratories). Experimental procedures were approved by the University of Illinois College of Medicine at Peoria IACUC review board. All methods were performed in accordance with the relevant guidelines and regulations. Mice were kept in a barrier room in 12 hours dark-light cycle, fed with regular chow, and water was available *ad libitum*. Leukemia was induced (n = 8 leukemic, n = 8 controls) as previously described [16] using CCL-119 cells (lymphoblasts from a pediatric acute lymphocytic leukemia patient, from ATCC). Blood samples were taken weekly via tail nicking, and RNA was extracted immediately after sampling using the QIAmp RNA Blood mini kit (Qiagen) according to the manufacturer’s protocol (S1 Fig). Three leukemic mice died on the 5th week of the experimental period. The following humane endpoints were used for immediate euthanasia: severe weight loss (>15% from starting weight), body condition score 2, ascites, moribund condition, dehydration. Body weight was measured twice a week.

After 5 weeks, anesthetized mice were euthanized via cervical dislocation. Anesthesia was performed with IsoThesia (Henry Schein Animal Health) using a vaporizer in an enclosed chamber until mice were recumbent. Anesthesia was tested by the lack of rear foot reflex. Small intestine was collected and immune cells were specifically extracted using the Lamina propria dissociation kit (Miltenyi biotech). Microscopic examination confirmed the absence of epithelial cells in cell preparations. Immune cell count was performed using Neubauer improved C-chip hemocytometers (InCyto).

### 3.2 RT-qPCR

RT-qPCR was performed on peripheral blood samples and on lymphocytes isolated from the GALT. IL-2, CD-3, -8, -45 PCRs were performed using the Quantitect primer assays (Qiagen) with the Quantitect RT-qPCR kit on a Rotor-Gene 6000 instrument. The 16S PCR was performed using the 530F (5’ GTG CCA GCM GCN GCG G 3’) - 806R (5’ GGA CTA CHV GGG TAT CTA AT 3’) primers. The reactions were incubated at 50 ^o^C for 30 minutes for reverse transcription, then at 95 ^o^C for 15 minutes for denaturation of DNA and activation of the polymerase. The reactions were then cycled 50 times at 94 ^o^C for 15 seconds, 50 ^o^C for 30 seconds and 72 ^o^C for 30 seconds. No template controls (NTC) were used in every run, and quantification of the 16S amplicons were performed by using a serial dilution of genomic DNA from *Escherichia coli* (Sigma). The 16S PCR was the only reaction which resulted in an amplification curve for NTC due to the inherent background amplification [17]. In this case, sample was counted positive if the Ct value of the sample was lower than CtNTC—5% of Ct_NTC_ [18]. Student’s t-test was used for statistical analyses, with significance level set to 0.05.

Metagenomics sequencing of the 16S amplicons was performed using a MiSeq instrument and was analyzed on MG-RAST (https://www.mg-rast.org). The 16S sequencing data is publicly available on MG-RAST under submission number MGP85109.

### 3.3 RNA-seq and data analysis

RNA-seq was performed on lymphocytes isolated from the GALT. The sequencing work was performed at the University of Illinois Roy Carver Biotechnology Center. Paired end libraries were constructed using the Ovation Solo RNA-seq system for mouse (Nugen). Sequencing was performed on a HiSeq 4000 instrument with 2x100 base pair read length. Raw and processed sequencing data has been uploaded to NCBI GEO (accession number GSE116234).

Sequencing reads have been mapped to the *Mus musculus* (GRCm38.p6) reference genome using Bowtie [19] with the following arguments: -a–m1 –n3 –l28 –e70 –p8. The normalization and statistical evaluation of differential gene expression has been performed using edgeR [20] with a p-value cut-off of 0.05 in RobiNA [21]. Differentially expressed genes with the top 100 log fold-change and with at least 50 reads were selected.

Functional annotation was performed using David [22]. Gene networks were constructed with GeneMania [23].

## Results

### 4.1 Cell count and expression analysis in the GALT in leukemia

**Fig 1:**
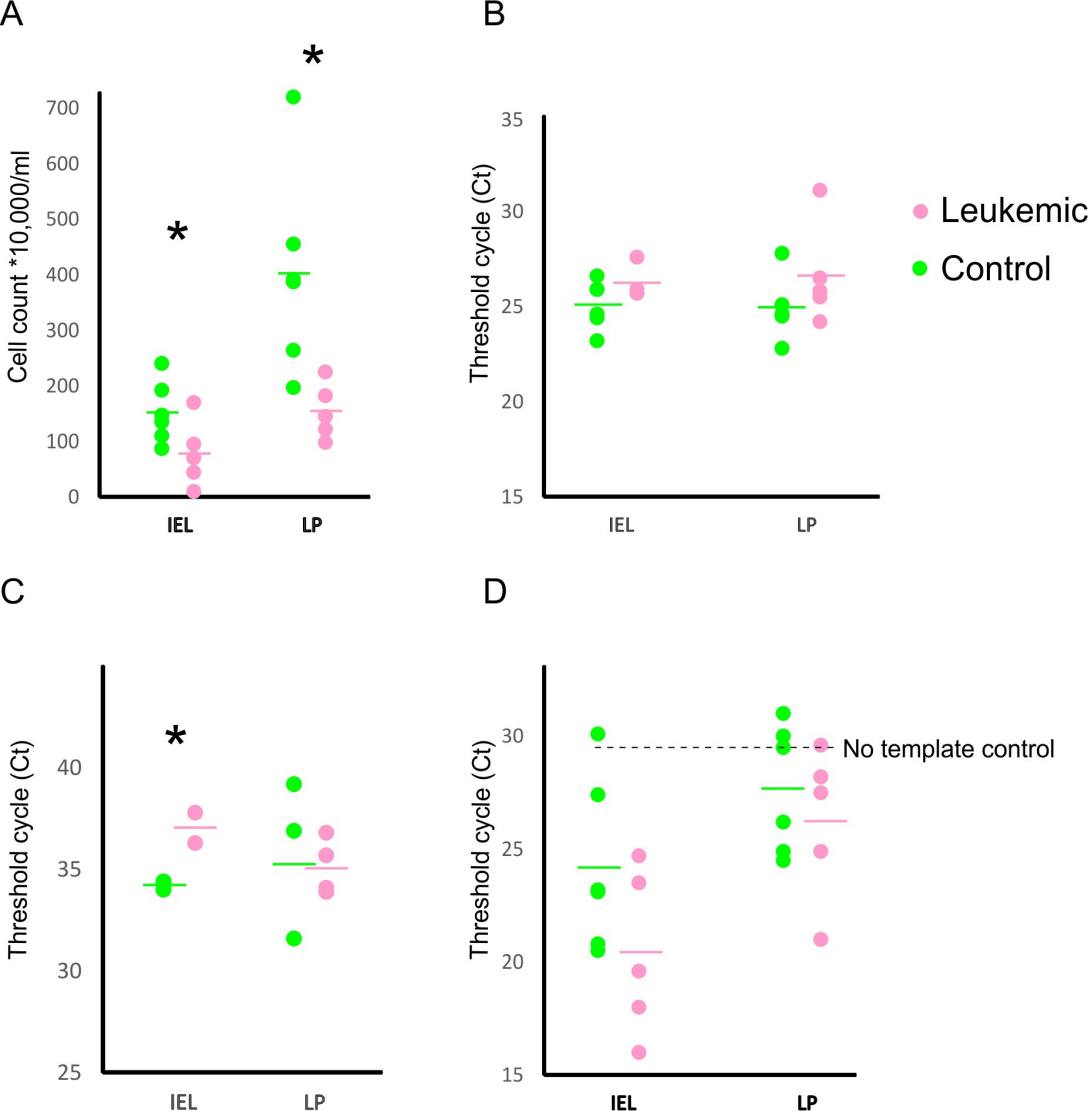
Alterations in the GALT in leukemia. Decreased number of lymphocytes were detected in leukemic mice (A) by hemocytometer both in the intraepithelial lymphocyte (IEL) population and lymphocytes in the lamina propria (LP), confirmed by CD45 RT-qPCR (B). The IL-2 expression level was significantly decreased in leukemic mice in IEL (C), and the GALT contained more bacteria in leukemic mice as shown with 16S RT-qPCR (D). Circles represent individual mice, horizontal lines show average values. Ct values are shown on the Y axis (B-D).

GALT is the largest immune organ in the body with a major role of containing the gut microbiota in the gastrointestinal tract, and therefore prevent the translocation of bacteria and other microbes. In order to evaluate the integrity of GALT in leukemia, we performed a cell count of lymphoid cells in GALT compartments. A significant decrease was found in the number of lymphoid cells both in the IEL (control/leukemic mean ± SD: 151.8 ± 55.9 / 77.8 ± 60.4) and LP (402.5 ± 181.8 / 154.4 ± 50.2) in leukemic mice (Fig 1A), indicating GALT structural damage.

In order to confirm the decrease in lymphoid cells, RT-qPCR targeting the universal leukocyte marker CD45 was performed. Similarly to cell count, it also detected a 2-fold decrease of leukocytes (Fig 1B) in IEL (Ct levels in control/leukemic mean ± SD: 25.1 ± 1.3 / 26.2 ± 0.9; 2^-ΔΔCt^ = 2.14) and a 3-fold decrease detected in LP (25 ± 1.8 / 26.6 ± 2.6; 2^-ΔΔCt^ = 3.03).

Because of the decrease of leukocytes in GALT in leukemia, we investigated whether interleukin-2 (IL-2) expression levels changed as well. IL-2 is an important part of the adaptive immune system and a major promoter of T-cell proliferation. In IEL, IL-2 levels showed a significant decrease (Ct levels in control/leukemic mean ± SD: 34.3±0.2 / 37.1±1, p<0.01), while in LP IL-2 levels were approximately equal (35.9±3.9 / 35.1±1.4, p = 0.36) between control and leukemic samples (Fig 1C).

We also measured CD8 expression levels in control and leukemic mice using RT-qPCR, as CD8^+^ cells play a major role in defending the host against invading pathogens. The results indicated a non-significant decrease in CD8 cells in leukemic animals in IEL (Ct values in leukemic / control ± SD: 28.7±1.4 / 24.4 ± 5.1, p = 0.16) and in LP (25.5 ± 4.47 / 24.5 ± 3.89, p = 0.36). IL-6 is an inflammatory cytokine secreted by T-cells and macrophages, and can play a role both in anti-inflammatory and pro-inflammatory processes. No significant differences were found between leukemic and control mice (Ct values in leukemic / control ± SD: IEL 32.1±0.5 / 34±2.9, LP 36.6±7.1 / 35±7.1).

The 16S rDNA is a universal marker present in all bacteria, and was used here for bacterial detection. Since the GALT is responsible to contain the gut microbiota, we used 16S rDNA levels to investigate whether the amount of bacteria differs in IEL and LP in leukemia. Leukemic mice showed a non-significant increase in the amount of bacteria both in IEL (Ct levels in control/leukemic mean ± SD: 24.2±3.8 / 20.4±3.7, p = 0.06) and LP (27.7±2.8 / 26.2±3.4, p = 0.23) (Fig 1D).

In summary, these measurements in the GALT indicated a structural impairment characterized by loss of immune cells via IL-2 mediated processes, which eventually led to an increase in bacterial levels in leukemic mice.

### 4.2 Expression changes in the blood using RT-qPCR

Blood samples were taken from leukemic and control mice once a week and RNA was extracted from blood for downstream analysis. The aim was to measure transcriptional changes throughout the duration of the experiment in levels of bacteria and inflammatory markers indicating BT.

**Fig 2:**
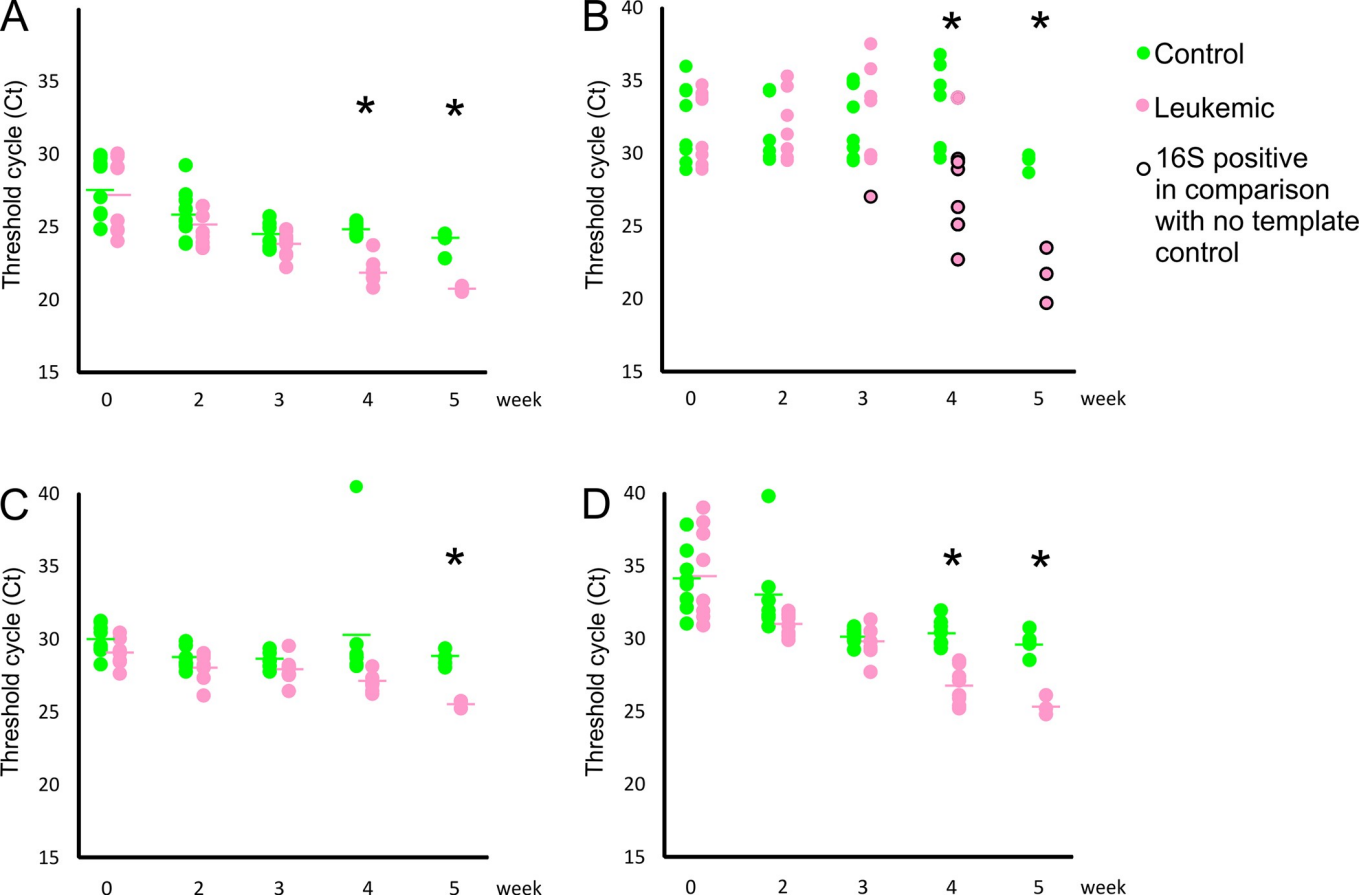
Alterations in the blood in leukemia over the experimental period. RT-qPCR indicated an increase in leukocyte levels by measuring CD45 expression by weeks 4 and 5 (A). Bacterial translocation was present in 87.5% of leukemic mice (0% in controls) by week 4, and all mice developed BSI by week 5 (B). RT-qPCR detected an increase in cytotoxic lymphocytes by measuring CD8 expression by week 5 (C) and increased TNF-α levels by weeks 4–5 (D).

In order to evaluate changes in leukocyte level, we performed CD45 RT-qPCR on blood samples taken from leukemic and control animals. By week 4, CD45 levels in leukemic animals exceeded controls, indicating elevated leukocyte count (Fig 2A, p<0.01)). Since we detected a decrease of CD45^+^ cells in the GALT, the increase of these cells in the blood may indicate an interrupted lymphocyte trafficking as observed in lymphomas [24].

As shown above, leukemia affects the intestinal lymphatic tissue and based on our hypothesis, this damage contributes to BSI. To test this hypothesis, we performed 16S RT-qPCR on blood samples taken from leukemic and control mice. By week 3, BSI started to appear (12.5% of leukemic animals) indicating BT. By week 4, 87.5%, and by week 5, 100% of leukemic animals (none of the controls) experienced BSI (Fig 2B, p<0.05, S2A Fig). Coefficient of variation was below 5% in both batches of control mice. Metagenomics sequencing of the 16S amplicons revealed a dominance of Proteobacteria and Firmicutes in blood samples (S2B Fig), similarly to clinical samples [4].

CD8 levels were also measured from blood as CD8 levels are elevated in pediatric ALL [25], and a significant increase was detected by week 5 in leukemic animals (Fig 2C, p<0.05). However, no significant differences were found in the CD3^+^ levels in the blood, although CD3^+^ cells help to activate CD8^+^ cells. Even though we detected an increase of CD3^+^ cells in the blood, the difference was not significant and may require a larger sample number to show this correlation (p = 0.06 on week 4, p = 0.25 on week 5).

Tumor necrosis factor-alpha (TNF-α) is a pleiotropic cytokine involved in inflammatory processes and anti-tumor responses, including hematological malignancies [26]. In addition, microbial metabolites modulate pathogen-induced TNF-α responses [27]. Therefore, we examined TNF-α expression changes in leukemia. A significant increase was observed in leukemic animals both on weeks 4–5 (p<0.01 on both weeks), indicating a BSI-induced TNF-α activation (Fig 2B–2D).

In summary, the measurements in the blood indicated the increase of inflammation and bacterial translocation as the disease progressed in leukemic animals.

### 4.3 RNA-seq analysis of lymphocyte migration in the GALT

**Fig 3:**
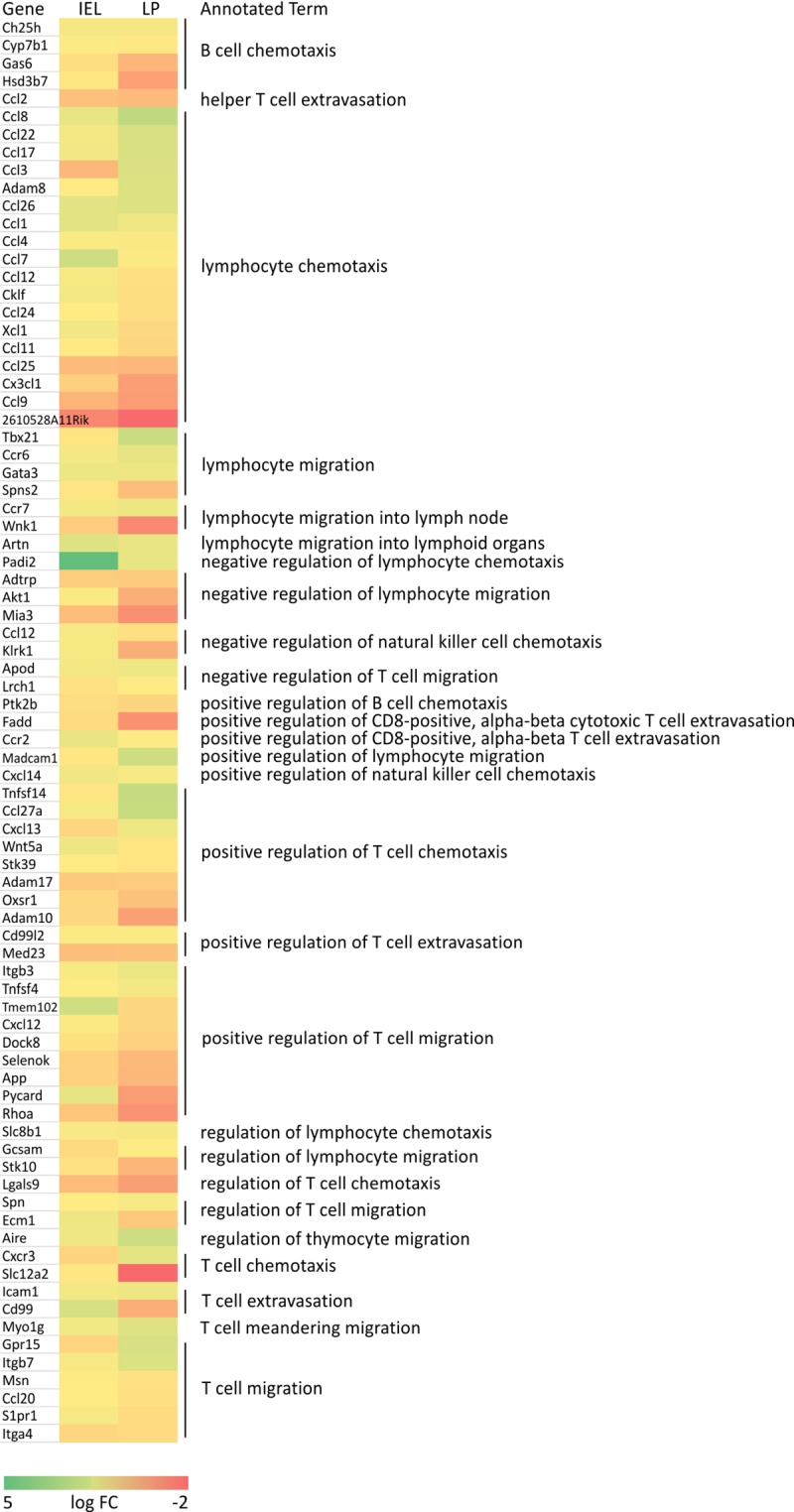
Logarithmic fold change (LogFC) values from RNA-seq of GALT lymphoid cells are shown in heat map for genes associated with lymphocyte migration.

Next, we aimed to investigate the expression levels of genes participating in lymphocyte migration as a cause of the reduced lymphocyte count using RNA-seq. In ALL, the exploitation of the physiological process of lymphocyte migration plays a role in infiltration of lymphocytes to distant organs, such as the small intestine [28]. The Padi2 gene, which negatively regulates lymphocyte chemotaxis, was the most highly expressed in leukemic mice compared to control (Fig 3). While Slc12a2 was the most underexpressed, representing a suppressed T-cell chemotaxis [29].

### 4.4 Whole exome RNA-seq analysis of GALT

**Fig 4:**
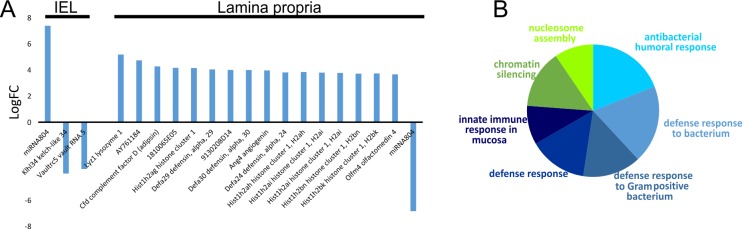
RNA-seq results identify differentially expressed genes in leukemic mice in IEL and LP (A). Functional annotation of differentially expressed genes in LP forms two major clusters: the histone cluster associated with chromatin modification (shades of green) and the antimicrobial cluster, associated with defense response to bacteria (shades of blue, B).

The goal of this step was to characterize and identify differentially expressed genes on the exome level. On average, 25.7M reads were obtained per sample. Differentially expressed genes were identified in leukemic mice both in IEL and in LP (Fig 4A).

#### 4.4.1 Differentially expressed genes in IEL

In IEL in leukemic mice, miRNA 804 was overexpressed. The function of this miRNA is currently unknown, although it may play an important role in intestinal barrier function and/or defensive functions against pathogens, as this miRNA was also differentially expressed (underexpressed) in LP in leukemic mice (Fig 4A).

Genes coding for Kelch-like protein 34 (Klhl34) and vault RNA 5 were underexpressed in the IEL from leukemic mice (Fig 4A). Kelch-like proteins are part of the ubiquitination process. The function of Klhl34 is not completely understood, but its underexpression has been associated with resistance to radiation and chemotherapy in cancer [30, 31]. Vault RNAs are considered to play a role in vault protein interactions, although their complete function is not known. Vault RNA5 was also found to be underexpressed in chronic lymphocytic leukemia [32] and in colorectal cancer [33].

#### 4.4.2 Differentially expressed genes in LP

In LP, 17 genes were differentially expressed in leukemia, 16 overexpressed and 1 underexpressed (Fig 4A). Two major clusters were found in overexpressed genes: the histone cluster and the antimicrobial cluster (Fig 4B).

Lysozyme 1, adipsin and defensin mRNAs were overexpressed in leukemic GALT (Fig 4A); they constitute an important part of mucosal immunity and are involved in defending the host from invading pathogens. Lysozyme 1 is an antimicrobial enzyme, and part of the innate immune system with particular efficacy against Gram-positive pathogens [34]. It also enhances repairs of the small intestine [34] and is overexpressed in ulcerative colitis [35]. Defensins are antimicrobial peptides, which disrupt bacterial membranes and is active against fungi and viruses. These molecules may also indirectly block BT by trapping bacteria in fibrils [36]. In addition, in combination with IL-18, defensins were shown to express antileukemic activity [37] and were applied in antimicrobial gene therapy [38].

The network of overexpressed antimicrobial genes indicate shared protein domains amongst these genes and other defensins and lysozymes (S3 Fig). This network includes metabolic regulators such as adiponectin (Adipoq) and α-lactalbumin (Lalba), and the latter also possesses bactericidal activity.

The histone cluster consists of genes responsible for nucleosome assembly and chromatin silencing. Overexpression of the histone 1 cluster indicates an increase in transcription suppression, and may lead to genomic instability and enhanced sensitivity to DNA damage [39]. Nonsense mutations have been detected in histone cluster 1 (HIST1H2AG), which may contribute to leukemogenesis [40]. Histone deacetylases are a group of enzymes, which remove acetyl groups from acetyl lysine on histones, allowing tighter DNA wrap to regulate gene expression [41]. These enzymes are commonly overexpressed in leukemias, and may be utilized as novel diagnostic and therapeutic targets [41]. The histone gene network (S4 Fig) reveals other repressive elements of histone regulations, such as the Sap18, part of the SIN-2 repressing complex or Zpf219, a transcription repressor. In addition, the two genes identified in the regulation of lymphocyte migration are also involved in the regulation of chromatin disassembly [29].

In summary, RNA-seq analysis of the GALT indicates impaired lymphocyte migration, and increased suppression of transcription through the histone 1 cluster, and elevated antimicrobial activity was also observed to eliminate translocating pathogens.

## Discussion

BSI is a severe and life-threatening complication commonly occurring in leukemia. A body of work points to the gut microbiota as the source of infection due to BT. In this work, we investigated the structure of the GALT, the mechanisms of BT and their contributions to the development of BSI in leukemia.

Our results showed that the GALT structure is severely altered in leukemia, at least in part due to the suppression of the IL-2 gene expression in IEL, which may also effect cytokine production in LP [42]. In addition, GALT damage may also be due to impairments in the process of lymphocyte migration [28] (Fig 3). This alteration then led to BT and subsequent BSI and inflammation, characterized by increased 16S rRNA, CD8^+^ cells and TNF-α levels.

Bacterial levels in IEL and LP showed a difference in leukemic mice compared to control, indicating that the GALT function of isolating bacteria from interior body parts may be hampered in leukemia. RNA-seq analysis identified several over- and underexpressed genes in GALT in leukemia, which can be potential therapeutic targets [30, 43]. This analysis also revealed a transcription repression and an increased antimicrobial activity in LP in response to BT, which, however, was not sufficient to prevent BSI in leukemia as shown by the presence of bacteria in blood.

There is an inherent limitation to use solely 16S RT-qPCR for bacterial detection, but strain-specific primers could not be applied due to limited starting material in this study. In addition, 16S PCR is prone to cross-contamination, but it is avoidable with strict guidelines we adhered to, such as the use of sterile supplies, and mechanical and chemical barriers [44].

Taken together, our results indicate that impairments in the GALT characterized by a decrease in lymphoid cells potentially lead to BT in ALL. This study also identified several genes, which may play a crucial role in the formation of BSI in GALT.

## Supporting information

S1 Fig Schematics of the experimental design. (TIF)

S2 Fig Quantification of the 16S amplicons showed an increase in copy numbers (A) in leukemic mice but not in controls, as measured by RT-qPCR using standard curve. Mean values are shown. High-throughput sequencing of the amplicons indicate an excess of Proteobacteria and Firmicutes (B). Bars represent 16S positive blood samples.
(TIF)

S3 Fig Defensin network with shared protein domains (brown lines) and co-expression (purple lines). Circles show function: purple: Defense response to bacterium, green: defense response to Gram positive bacterium. Striped genes are overexpressed in current study.
(TIF)

S4 Fig Histone network. Brown lines: Shared protein domains (Interpro), pink lines: physical interactions. Striped genes are overexpressed in current study.
(TIF)
