# Supplementary figures and images for: Bacterial translocation in acute lymphocytic leukemia
Source: PLoS One. 2019 Apr 1;14(4):e0214526. doi: 10.1371/journal.pone.0214526 (PMC6443231; doi:10.1371/journal.pone.0214526)

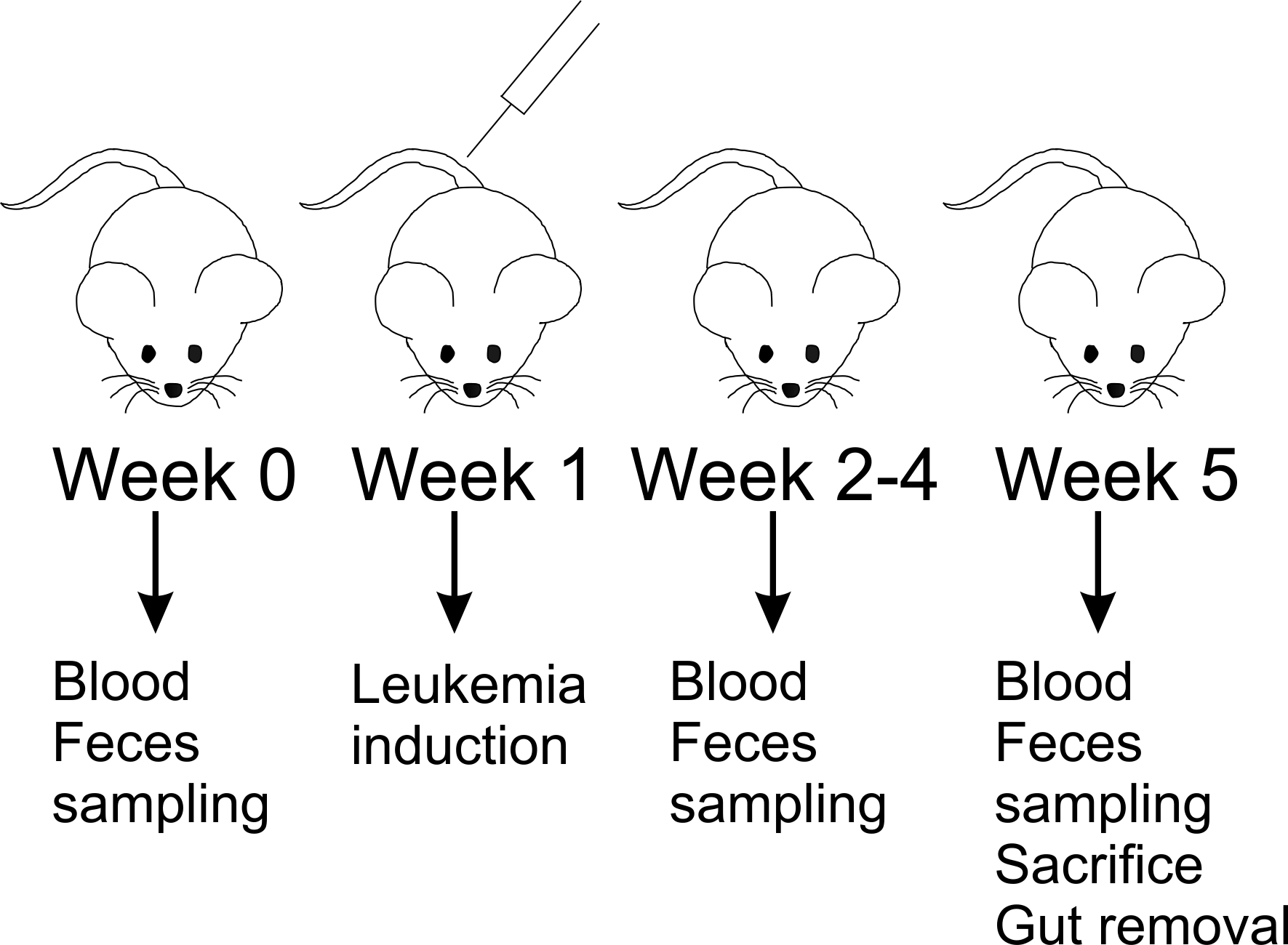

Supplement: S1 Fig — (TIF) [file pone.0214526.s001.tif]

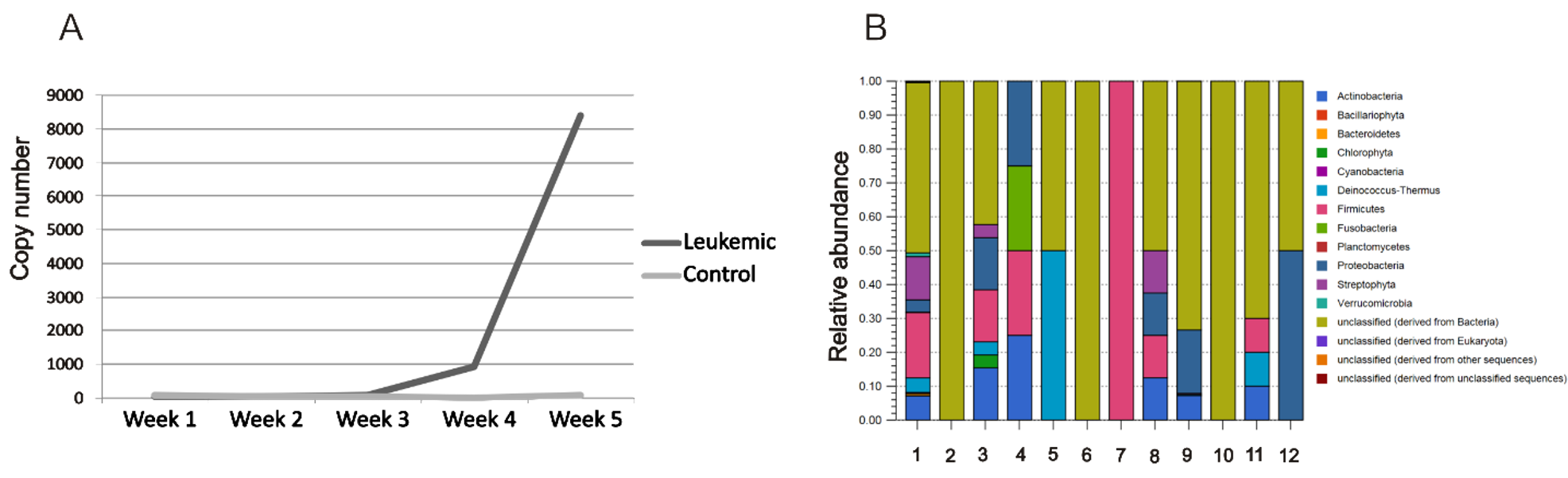

Supplement: S2 Fig — Mean values are shown. High-throughput sequencing of the amplicons indicate an excess of Proteobacteria and Firmicutes (B). Bars represent 16S positive blood samples. (TIF) [file pone.0214526.s002.tif]

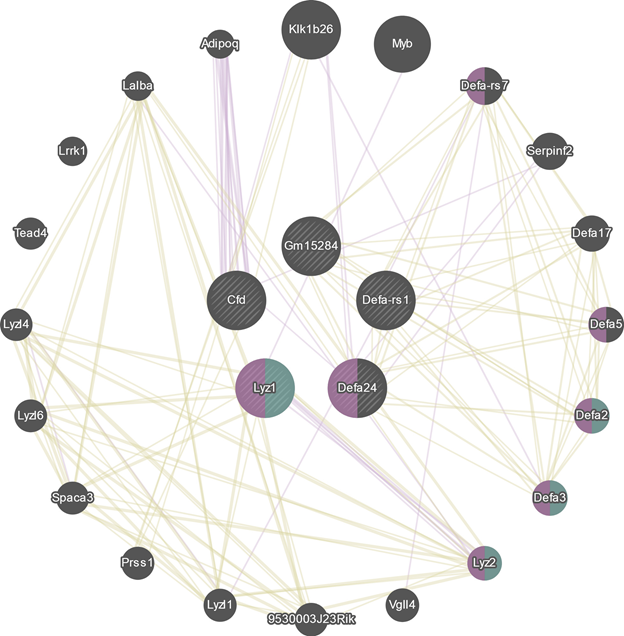

Supplement: S3 Fig — Circles show function: purple: Defense response to bacterium, green: defense response to Gram positive bacterium. Striped genes are overexpressed in current study. (TIF) [file pone.0214526.s003.tif]

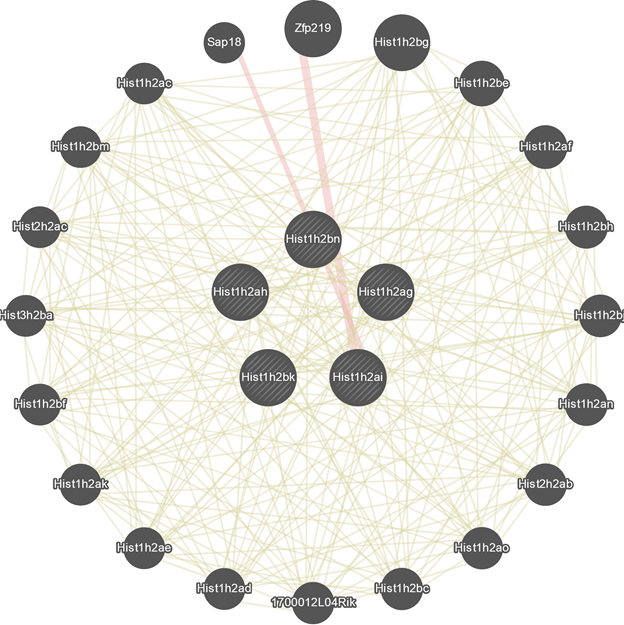

Supplement: S4 Fig — Brown lines: Shared protein domains (Interpro), pink lines: physical interactions. Striped genes are overexpressed in current study. (TIF) [file pone.0214526.s004.tif]
